# Modelling the potential acute and post-acute burden of COVID-19 under the Australian border re-opening plan

**DOI:** 10.1186/s12889-022-13169-x

**Published:** 2022-04-14

**Authors:** Mary Rose Angeles, Sithara Wanni Arachchige Dona, Huong Dieu Nguyen, Long Khanh-Dao Le, Martin Hensher

**Affiliations:** 1 Institute for Health Transformation, Faculty of Health, Deakin University, 221 Burwood highway, Burwood, Victoria 3125 Australia; 2 Deakin Health Economics, School of Health and Social Development, Faculty of Health, Deakin University, 221 Burwood highway, Burwood, Victoria 3125 Australia; 3 Health economics Division, School of Public Health and Preventive Medicine, Monash University, Melbourne, Victoria Australia; 4 Menzies Institute for Medical Research, University of Tasmania, Medical Science Precinct, 17 Liverpool Street, Hobart, Tasmania 7000 Australia

**Keywords:** Burden of disease, Disability-adjusted life years, DALY, Coronavirus, Covid-19, Long COVID, Post-COVID, Post-acute COVID, Australia

## Abstract

**Background:**

Concerns have grown that post-acute sequelae of COVID-19 may affect significant numbers of survivors. However, the analyses used to guide policy-making for Australia’s national and state re-opening plans have not incorporated non-acute illness in their modelling. We, therefore, develop a model by which to estimate the potential acute and post-acute COVID-19 burden using disability-adjusted life years (DALYs) associated with the re-opening of Australian borders and the easing of other public health measures, with particular attention to longer-term, post-acute consequences and the potential impact of permanent functional impairment following COVID-19.

**Methods:**

A model was developed based on the European Burden of Disease Network protocol guideline and consensus model to estimate the burden of COVID-19 using DALYs. Data inputs were based on publicly available sources. COVID-19 infection and different scenarios were drawn from the Doherty Institute’s modelling report to estimate the likely DALY losses under the Australian national re-opening plan. Long COVID prevalence, post-intensive care syndrome (PICS) and potential permanent functional impairment incidences were drawn from the literature. DALYs were calculated for the following health states: the symptomatic phase, Long COVID, PICS and potential permanent functional impairment (e.g., diabetes, Parkinson’s disease, dementia, anxiety disorders, ischemic stroke). Uncertainty and sensitivity analysis were performed to examine the robustness of the results.

**Results:**

Mortality was responsible for 72-74% of the total base case COVID-19 burden. Long COVID and post-intensive care syndrome accounted for at least 19 and 3% of the total base case DALYs respectively. When included in the analysis, potential permanent impairment could contribute to 51-55% of total DALYs lost.

**Conclusions:**

The impact of Long COVID and potential long-term post-COVID disabilities could contribute substantially to the COVID-19 burden in Australia’s post-vaccination setting. As vaccination coverage increases, the share of COVID-19 burden driven by longer-term morbidity rises relative to mortality. As Australia re-opens, better estimates of the COVID-19 burden can assist with decision-making on pandemic control measures and planning for the healthcare needs of COVID-19 survivors. Our estimates highlight the importance of valuing the morbidity of post-COVID-19 sequelae, above and beyond simple mortality and case statistics.

**Supplementary Information:**

The online version contains supplementary material available at 10.1186/s12889-022-13169-x.

## Background

In response to the coronavirus disease 2019 (COVID-19) pandemic, Australian governments implemented a number of strict strategies, including international border closure, intermittent interstate border closure and lockdowns, effectively aiming to achieve COVID-19 zero [1]. Australia’s first pandemic measure began on the 15th of March 2020, when international arrivals were mandated to self-quarantine for 14 days. The government also imposed social distance rules and restrictions on non-essential gatherings 3 days later. The closure of non-essential businesses such as places of worship, gyms, recreational venues were also mandated on the 21st of March 2020 [2]. Tighter measures were implemented on the 30th of March 2020 where “stay at home” orders were imposed except for essential activities such as medical care, exercise, food shopping. International travel has been severely restricted, not only through strict arrival caps and quarantine requirements for incoming travellers but also by federal restrictions on the ability of Australian citizens and residents to leave the country without special exemptions for travel. Travel between states was significantly reduced due to periodic interstate border restrictions (and, indeed, restrictions on internal travel within some states). Since early 2020, the precise public health measures have differed between each state and territory in Australia; restrictions have been periodically tightened and relaxed depending on the levels of transmission, case numbers and perceived risk levels [2–4].

While these measures have been effective in reducing the number of COVID-19 cases and deaths compared to other nations, Australia has suffered significant economic losses as a result [1]. Vaccination is currently the central strategy for returning to pre-pandemic social lifestyles and economic activity. Although COVID-19 vaccines cannot fully contain the spread of transmission, they offer significant protection from more severe health impacts of COVID-19 [1]. The National Cabinet (comprising the federal Prime Minister and the Premier / First Ministers of each state and territory) has agreed on a national re-opening plan for international borders and relaxing restrictions [5], and Australia is now transitioning from pre-vaccination to post-vaccination setting and moving towards a “living with COVID-19 strategy”.

To support the development of this plan, the Doherty Institute was commissioned by the National Cabinet to model the impact of different re-opening scenarios, based on vaccination coverage, public health and social measures (PHSM), the effectiveness of testing, trace, isolation, quarantine (TTIQ), with consideration of seeding infection growth [6]. The Doherty modelling provided detailed outputs on new cases, hospitalisation and mortality but did not estimate the potential longer-term consequences of post-acute COVID-19 sequelae from these re-opening strategies. ‘Long COVID’ is referred to as the extended and more complex course of COVID-19 as a multisystem disease associated with a range of symptoms [7]. In addition, there is increasing evidence of organ impairment and late complications in some COVID-19 survivors [7–9]. This raises concerns that some patients will experience long-term or permanent disability following COVID-19, and historical evidence of long-term sequelae following viral pandemics also suggests that we should be on our guard for the potential long-term impacts among survivors [7].

To better understand the dynamics and relative impact of COVID-19, the aim of our model is to supplement the Doherty modelling by estimating the full burden of COVID-19 in Australia’s border re-opening strategy using disability-adjusted life years (DALYs), paying particular attention to post-acute consequences and the potential impact of permanent functional impairment as a consequence of COVID-19. Readers should note that the authors of this paper were not involved in the Doherty Institute modelling exercise, and our modelling results should not be interpreted as bearing the endorsement of the Doherty Institute team.

## Methods

A decision tree was developed based on the European Burden of Disease Network (Burden-EU) protocol guideline and consensus model [10, 11] to estimate the DALY loss from the modelled COVID-19 cases of Australia’s national roadmap to re-opening [6].

### Model structure

**Fig. 1:**
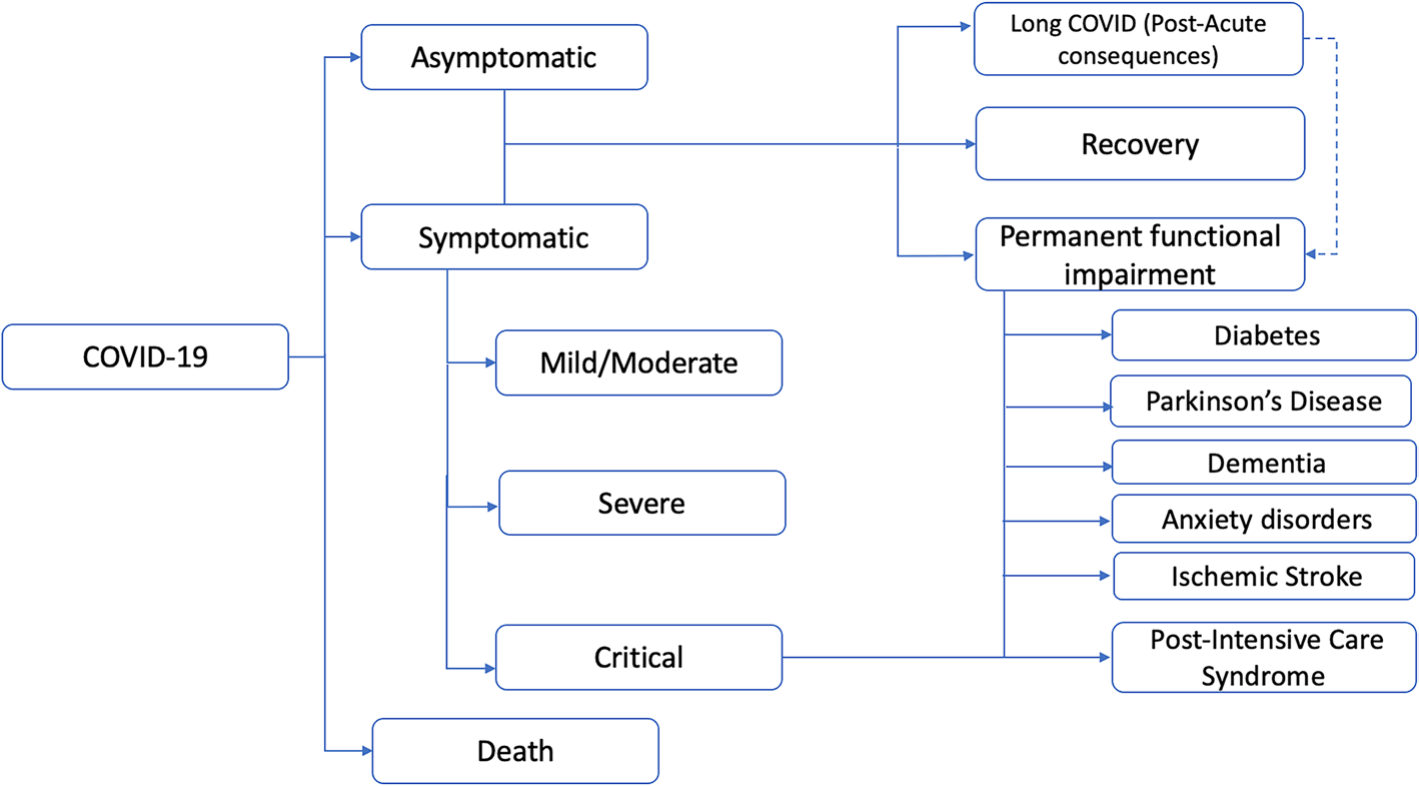
COVID-19 outcome model. Source: Adapted from Wyper et al .[10] under the Creative Commons Attribution License (CC BY)

The model in this study was developed in Microsoft Excel 2016 (see Additional file 1 for the DALY COVID-19 model) [10, 11]. Figure 1 presents the COVID-19 outcome model adopted in this study [10, 11]. Patients infected with COVID-19 were simulated in the model and could progress to various health states: asymptomatic, symptomatic (acute), or death [10, 11]. “Asymptomatic” refers to when a patient contracted the virus but does not show any symptoms [10, 11]. In the symptomatic health state, “acute cases” may experience mild/moderate symptoms but are not hospitalised, while “severe” symptoms require hospitalisation or a critical condition managed in the intensive care unit (ICU). Asymptomatic and symptomatic patients may experience a full recovery, Long COVID, or a permanent functional impairment [10, 11]. Long COVID patients experience longer-term symptoms after an acute COVID-19 infection and may not recover fully for some time. Over time, those with Long COVID either fully recover or develop permanent functional impairment. Permanent functional impairments included diabetes, Parkinson’s disease, dementia, anxiety disorders, and ischemic stroke. Patients managed in ICU may recover fully or develop post-intensive care syndrome (PICS). PICS is defined as “new or worsening impairments in physical, cognitive, or mental health status arising after critical illness and persisting beyond acute care hospitalisation” [12]. It is assumed that patients in the state of permanent functional impairment could not transition back to other post-acute recovery states.

### Disability-adjusted life years

Disability-adjusted life years (DALYs) were estimated by summing the life years lost due to premature mortality (YLL) and the years lived with disability (YLD) primarily using an incidence-based approach [10, 11].

$$\mathrm{DALY}=\mathrm{YLL}+\mathrm{YLD}$$

YLL is calculated from the COVID-19 mortality statistics (M) and average life expectancy (LE), presented into a 10-age group band generated from public national data sources [6, 13].

$$\mathrm{YLL}=\mathrm{M}\times \mathrm{LE}$$

YLD for acute COVID-19, Long COVID and PICS (YLDinc) was calculated by multiplying the number of COVID-19 cases (N), the average duration of health state until recovery or death (D), and disability weight (DW). The disability weight accounts for the extent of health loss associated with the specific health outcomes, ranging from 0 to 1 (0 = no impact or having full health, 1 = occurrence of death) [14].

$$\mathrm{YLDinc}=\mathrm{N}\times \mathrm{D}\times \mathrm{D}\mathrm{W}$$

Some COVID-19 survivors have developed permanent illness or disability (distinct from Long COVID) [7–9]. However, the evidence on permanent impairment is less robust than that for acute, Long COVID and PICS. We, therefore, separately illustrate some of the potential for long-term disability due to COVID-19. Conservatively, we only include the incidence of certain permanent functional impairments post-COVID most commonly observed in large cohort studies (e.g., new onset of diabetes, Parkinson’s disease, dementia, anxiety disorders, and ischemic stroke) [8, 9]. To estimate the DALYs associated with these conditions, we sourced DALYs per person with these conditions from the 2019 Australian Burden of Disease study (Table 2) [15].

The results of this model are presented in three scenarios. The base case DALYs consist of the mortality and morbidity impact of acute COVID-19, Long COVID and PICS. “Total Burden One” presents the base case result plus the potential impact of all listed permanent functional impairments. “Total Burden Two” *excludes* diabetes as a permanent impairment.

### Data inputs

The data were obtained from the literature, official statistics and Doherty’s modelling report [6], as updated in September 2021 and is further described in the following sections. Tables 1 and 2 present the summary of all the model inputs obtained from the publicly available sources. Ethics approval was not required as this study analysed publicly available data.

**Table 1:** Model inputs

Health States	Rates	Duration (Sensitivity Analysis)	DW (Uncertainty Analysis)
Death	Reported in Table ES1, ES2, 2.3 and 2.4 from Doherty Modelling report [6]. The rates of Australian total COVID-19 related death as of 03 October 2021 was employed to present deaths in a 10-age group band [16].	Nil	Nil
Asymptomatic	Not considered	Nil	Nil
Moderate	Calculated based on the reported symptomatic infections minus the Ward admission and ICU admission reported in Table ES1, ES2, 2.3 and 2.4 from Doherty Modelling report [6].	14 days [17]	0.051 (0.032 – 0.074) a [10, 11]
Severe	Reported in Table ES1, ES2, 2.3 and 2.4 from Doherty Modelling report [6].	14 days [17]	0.133 (0.088 – 0.190) a [10, 11]
Critical	Reported in Table ES1, ES2, 2.3 and 2.4 from Doherty Modelling report [6].	14 days [17]	0.655 (0.579 – 0.727) a [10, 11]
Post-acute consequences (ONS)	Start at 25.91% (23.2 to 29.0%) 2 weeks after initial COVID infection [18]^a^ See notes below on how we compute for the COVID-19 survivors noted in this calculation.	14 days to 2 years (assumed)	0.219 (0.148-0.308) [10, 11]
Post-acute consequences (NSW)	Start at 33.60% (33.0 to 34.0%) 2 weeks after initial COVID infection [19] See notes below on how we compute for the COVID-19 survivors noted in this calculation.	14 days to 2 years (assumed)	0.219 (0.148-0.308) [10, 11]
Post-Intensive Care Syndrome (PICS)	90.6% of ICU survivors [20] See notes below on how we compute for the COVID-19 survivors noted in this calculation.	14 days (to remaining lifetime expectancy) [21, 22]	0.224 (0.151-0.312)^a^ (a) [23, 24]

*PICS* Post Intensive Care Syndrome, *NSW* New South Wales, *ONS* Office of National Statistics, ^a^Used in uncertainty analysis using beta-distribution; a Symptomatic atrial fibrillation and flutter as proxy for PICS, refer to Additional file 2 for the calculation

**Table 2:** Data used in the permanent disability health states

Permanent Disability	Incidence Rate	Population of Interest	Duration	Australian Incidence	Australian DALYS
Diabetes	2.8% (2.6 to 3.1%) a [8]	COVID-19 survivors who were hospitalised (age 30 and over)	140 days following hospital admission	69,042.78 [15]	186,528.44 [15]
Parkinson’s disease	0.11% (0.08 to 0.14%) a [9]	COVID-19 survivors regardless of hospitalisation status (age greater than 10 years old)	6 months post COVID diagnosis	6598.01 [15]	38,742.45 [15]
Dementia	0.67% (0.59 to 0.75%) a [9]	COVID-19 survivors regardless of hospitalisation status (age greater than 10 years old)	6 months post COVID diagnosis	43,968.58 [15]	154,293.14 [15]
Anxiety Disorders	7.11%(6.82 to 7.41%) a [9]	COVID-19 survivors regardless of hospitalisation status (age greater than 10 years old)	6 months post COVID diagnosis	188,749.60 [15]	139,107.98 [15]
Ischaemic Stroke	0.76% (0.68 to 0.85%) a [9]	COVID-19 survivors regardless of hospitalisation status (age greater than 10 years old)	6 months post COVID diagnosis	17,984.10 [15]	114,238.13 [15]

^a^Used in uncertainty analysis using beta-distribution

### Doherty COVID-19 modelling

The Doherty Institute COVID-19 model [6] was developed to inform Australia’s national COVID-19 re-opening plan; it estimated the potential health and health system impacts of COVID-19 after eligible Australians achieve different coverage levels of full doses of COVID-19 vaccines (i.e. 50-80%). Transmission potential of COVID-19 delta variant, different bundles of public health and social distancing measures (PHSM), the efficacy of test-trace-isolate-quarantine activities (TTIQ), and the seeding infection rate (“*initial number of daily cases present in the population at a given vaccination threshold”*) were included in the Doherty analysis. Doherty model outputs over the first 180 days were reported in detail [6], enabling us to calculate the potential DALY burden for each strategy and illustrate the likely DALY burden arising from the post-acute consequences of COVID-19. The results and the analysis of our model are generated based on the most applicable hypothetical Doherty scenarios, given actual developments towards re-opening to date across Australia. All other scenarios were also presented and calculated in the Additional file 1 and Additional file 2: Table 1.

- Scenario 2C: Outbreaks seeded with 1000 to 4500 cases given partially effective TTIQ. The community has achieved COVID-19 vaccination coverage of 70% while maintaining low PHSM.
- Scenario 2D: Outbreaks seeded with 1000 to 4500 cases given partially effective TTIQ. The “*medium PHSMs are overlaid between the 70 and 80% coverage thresholds with reversion to low PHSMs thereafter”.*
- Scenario 3B: Outbreaks seeded with 300-1000 cases given partially effective TTIQ. The community has achieved COVID-19 vaccination coverage of 80% with baseline PHSM.
- Scenario 3C: Outbreaks seeded with 1000 to 4500 cases given partially effective TTIQ. The community has achieved COVID-19 vaccination coverage of 80% with baseline PHSM.

The full definition of TTIQ and different levels of PHSM are reported elsewhere [6, 25]. Briefly, due to the high volume of cases, expected delays in TTIQ responses are noted for “partial” TTIQ [6]. No “stay-at-home” orders, but low-density requirements (2 sqm rule) are imposed for “baseline” PHSM. Social distancing rules are still mandated, but retail trade and travel restrictions are not imposed [6, 25]. Rules mandated for baseline are also similar for “low” PHSM, however, there are some limitations in recreational, retail and workplace capacity under “low” PHSM [6, 25]. Under medium PHSM, “stay-at-home” orders are imposed unless for work, study and essential activities. However, work from home is recommended when possible. Schools, childcare and indoor recreational venues are closed. Intra and interstate travel are not allowed under medium PHSM [6, 25].

### Asymptomatic Health state

According to a systematic review and meta-analysis, approximately 17% (95%CI:14-20%) of total cases are asymptomatic [26]. However, the results of asymptomatic cases are not presented in the Doherty’s model, and therefore we did not include asymptomatic cases in our calculation.

### Symptomatic Health state

COVID-19 cases were obtained from the Doherty Modelling Interim Report to the national Cabinet (17th September 2021) [6], and a period of 14-days was conservatively used for the recovery duration for acute-COVID-19 state [17].

### Post-acute consequences

Our model assumed that Long COVID symptoms start directly after the symptomatic phase. Briefly, Long COVID refers to those patients experiencing any of the following symptoms: persistent fever, headache, muscle ache, weakness/tiredness/fatigue, nausea/vomiting, abdominal pain, diarrhoea, sore throat, cough, shortness of breath, loss or change of taste, loss or change of smell runny nose and chest pain post COVID-19 infection to reflect the symptoms observed in the studies of Liu et al. [19] and UK ONS cohort study [18]. Given the United Kingdom (UK) evidence that some patients still report Long COVID over 12 months after infection [18], our model assumed that Long COVID could potentially last up to 2 years. Due to the lack of longitudinal data regarding the length of Long COVID, we extrapolated available data on numbers of Long COVID cases over time from the UK Office of National Statistics (ONS) [18] and a population-based cohort study in New South Wales (NSW) [19] until it reached 0% using a fitted decay function [27]. This estimate was only applied to COVID-19 survivors. Moreover, the proportion of people with Long COVID also developing permanent functional impairment is not yet known and therefore this pathway was not included in this analysis.

According to a large case-control study in the UK, fully vaccinated individuals appear less likely to experience Long COVID following “breakthrough” infection compared with their unvaccinated counterparts (odds ratio = 0.51, 95%CI:0.32-0.82) [28]. This OR was converted to relative risk using the Cochrane formula [29] and was then applied in the vaccinated cohort.

### Permanent functional impairment

Evidence in the literature reported a wide range of permanent functional impairments after contracting COVID-19 [8, 9, 30–36]. To quantify the incidence of permanent disability post COVID-19, we considered conditions for which an incidence rate was available, and which also have an equivalent Australian DALY values sourced from the global burden of disease studies [15]. We used data from two large studies that investigated these conditions [8, 9]. A large US cohort study indicated the incidence of 0.11% (95%CI:0.08-0.14) for Parkinson’s disease, 0.67% (95%CI:0.59-0.75) for dementia, 7.11% (95%CI:6.82-7.41) for anxiety disorders and 0.76% (95%CI:0.68-0.85) for ischemic stroke within 6 months post-COVID-19 [9]. For diabetes, data from a large cohort study in the UK found that 2.83% (95%CI:2.57-3.12) of hospitalised patients were diagnosed with diabetes (type-1 or-2) over a mean follow-up of 140 days [8]. However, it is arguable whether diabetes was an undetected pre-existing condition or whether COVID-19 induced type-1 or type-2 diabetes [37, 38]. Thus, we presented our DALY calculation with (“Total Burden One”) and without the impact of diabetes (“Total Burden Two”).

### Post-intensive care syndrome

We also included the debilitating effects often seen after ICU admission, commonly referred to as post-intensive care syndrome (PICS) [20]. The morbidity of ICU survivors was kept separate from those with Long COVID as ICU survivors may experience “mid- long-term morbidities related to the critical illness” which is often reflected in three components: physical, mental health and cognitive impairment [20]. The only cohort study that investigated PICS in the COVID-19 population found that 90.60% of ICU survivors had PICS [20]. Unfortunately, there are no existing disability weights for PICS. A QALY utility score of 0.75 (95%CI: 0.63–0.84) was available for ICU COVID-19 survivors 12-16 weeks post discharge [39], hence a comparable illness with a similar utility score was assumed. We selected atrial fibrillation and flutter which has a utility score of 0.75 [23] and a disability weight of 0.22 (95%CI: 0.15–0.31) [24]. A lifetime duration was assumed for PICS reflecting study findings that followed ICU survivors over 5-years and 10-years [21, 22].

### Uncertainty analysis and pilot model testing

Uncertainty analyses were undertaken to propagate parameter uncertainty (i.e. sampling error) from the input parameters to the final model outputs. Monte Carlo simulation with 2000 iterations via the add-in tool Ersatz (Ersatz, Version 1.35) was used [40]. Estimates of DALYs were presented with 95% uncertainty intervals (95% UI).

The pilot testing of the model was conducted using actual observed Australian COVID-19 cases, hospitalisations and deaths data from January 2020 to January 2021. In this pilot, we have conducted different sensitivity analyses on rates of acute health states, post-acute rates, and a 28-day recovery period from the acute health state to explore whether these input parameters have impacted the robustness of the results (See Additional file 3). Findings showed only a very small change in DALYs. Therefore, we have not included these sensitivity analyses in the current study. In this study, we have instead conducted a sensitivity analysis to investigate the impact of changes in the rates of post-acute consequences, using NSW Long COVID data points starting at 34% at week three and 0% at week 104, in addition to the UK ONS data points [19].

## Results

Tables 3, 4 and 5 (See Additional file 4 for full results and corresponding 95%CIs) show the distribution of health states contributing to the total morbidity and DALY burden using the ONS data points. In all scenarios, the mortality impact (YLL) is the largest contributor to the base case DALY burden (72-74%), followed by the morbidity impact of Long COVID (19-22%). However, including the potential for permanent functional impairment over a lifetime had a much bigger impact. Once included, permanent disability would now dominate the total DALY burden (51-55%) or 80-82% of total morbidity impact, followed by YLL from mortality (32-36%), and Long COVID (9-10%). In contrast, mortality drove the overall burden of disease from COVID-19 in the first year of the pandemic in Australia, causing some 82% of the base case DALYs lost or 58% of Total DALY Burden One, followed by the morbidity impact of permanent disability (29%). Our results for 2020 are very similar to those recently obtained by the Australian Institute of Health and Welfare (AIHW) [41]. The differences in the composition of burden between year one and the Doherty scenarios reflect the significant reductions in mortality due to widespread vaccination.

**Table 3:** Estimated DALY burden for Doherty model scenarios, Base Case (no permanent disability)

DALY Burden Estimates - Base Case										
	Estimated DALY loss for each health state					Share of DALY loss for each health state				
Doherty Scenario	2C	2D	3B	3C	2020 Actual	2C	2D	3B	3C	2020 Actual
Deaths (no)	1524	948	6402	6719	909	n/a	n/a	n/a	n/a	n/a
COVID cases (no)	246,399	156,799	914,357	968,154	28,696	n/a	n/a	n/a	n/a	n/a
Mortality (YLL)	15,912	9898	66,844	70,154	7263	72.6%	72.3%	74.3%	73.8%	82.0%
Non-fatal YLD:										
Acute	558	353	2069	2213	69	2.5%	2.6%	2.3%	2.3%	0.8%
Long COVID (ONS)	4665	2981	17,467	18,482	565	21.3%	21.8%	19.4%	19.4%	6.4%
PICS	779	462	3578	4198	958	3.6%	3.4%	4.0%	4.4%	10.8%
Permanent	n/a	n/a	n/a	n/a	n/a	n/a	n/a	n/a	n/a	n/a
Total non-fatal YLD	6003	3797	23,114	24,843	1592	27.4%	27.7%	25.7%	26.2%	18.0%
Total DALYs (fatal and non-fatal)	21,915	13,695	89,958	94,998	8855	100%	100%	100%	100%	100%

*Notes*: *no* Number, *YLL* Years of life lost, *YLD* Years lived with disability, *DALYS* Disability adjusted life years, *Long COVID ONS* result using ONS data points, *PICS* Post-Intensive care syndrome and Permanent = Permanent functional impairment, Base case = excluded the burden of permanent disability, results are referring to the combined mean burden of both vaccinated and unvaccinated individuals, *n/a* not applicable

**Table 4:** Estimated DALY burden for Doherty model scenarios, Total Burden One (permanent disability including diabetes)

DALY Burden Estimates – Total Burden One										
	Estimated DALY loss for each health state					Share of DALY loss for each health state				
Doherty Scenario	2C	2D	3B	3C	2020 Actual	2C	2D	3B	3C	2020 Actual
Deaths (no)	1524	948	6402	6719	909	n/a	n/a	n/a	n/a	n/a
COVID cases (no)	246,399	156,799	914,357	968,154	28,696	n/a	n/a	n/a	n/a	n/a
Mortality (YLL)	15,912	9898	66,844	70,154	7263	32.4%	32.2%	36.1%	35.5%	58.3%
Non-fatal YLD:										
Acute	558	353	2069	2213	69	1.1%	1.2%	1.1%	1.1%	0.5%
Long COVID (ONS)	4665	2981	17,467	18,432	565	9.5%	9.7%	9.4%	9.3%	4.5%
PICS	779	462	3578	4198	958	1.6%	1.5%	1.9%	2.1%	7.7%
Permanent	27,131	17,009	95,215	102,537	3611	55.3%	55.4%	51.4%	51.9%	29.0%
Total non-fatal YLD	33,134	20,807	118,329	127,380	5203	67.6%	67.8%	63.9%	64.5%	41.7%
Total DALYs (fatal and non-fatal)	49,046	30,705	185,174	197,534	12,467	100.0%	100%	100%	100%	100%

*Notes*: *no* Number, *YLL* Years of life lost, *YLD* Years lived with disability, *DALYS* Disability adjusted life years, *Long COVID ONS* result using ONS data points, *PICS* Post-Intensive care syndrome and Permanent = Permanent functional impairment, Total burden one = overall burden including all the permanent disability, results are referring to the combined mean burden of both vaccinated and unvaccinated individuals, *n/a* Not applicable

**Table 5:** Estimated DALY burden for Doherty model scenarios, Total Burden Two (permanent disability excluding diabetes)

DALY Burden Estimates – Total Burden Two										
	Estimated DALY loss for each health state					Share of DALY loss for each health state				
Doherty Scenario	2C	2D	3B	3C	2020 Actual	2C	2D	3B	3C	2020 Actual
Deaths (no)	1524	948	6402	6719	909	n/a	n/a	n/a	n/a	n/a
COVID cases (no)	246,399	156,799	914,357	968,154	28,696	n/a	n/a	n/a	n/a	n/a
Mortality (YLL)	15,912	9898	66,844	70,154	7263	37.0%	36.7%	40.8%	40.2%	59.3%
Non-fatal YLD:										
Acute	558	353	2069	2213	69	1.3%	1.3%	1.3%	1.3%	0.6%
Long COVID (ONS)	4665	2981	17,467	18,432	565	10.8%	11.1%	10.7%	10.6%	4.6%
PICS	779	462	3578	4198	958	1.8%	1.7%	2.2%	2.4%	7.8%
Permanent	21,113	13,263	73,804	79,345	3401	49.1%	49.2%	45.1%	45.5%	27.7%
Total non-fatal YLD	27,116	17,060	96,918	104,188	4992	63.0%	63.3%	59.2%	59.8%	40.7%
Total DALYs (fatal and non-fatal)	43,028	26,958	163,763	174,343	12,256	100%	100%	100%	100%	100%

*Notes*: *no* number, *YLL* Years of life lost, *YLD* Years lived with disability, *DALYS* Disability adjusted life years, *Long COVID ONS* result using ONS data points, *PICS* Post-Intensive care syndrome and Permanent = Permanent functional impairment, Total burden two = overall burden including all the permanent disability excluding diabetes, results are referring to the combined mean burden of both vaccinated and unvaccinated individuals, *n/a* not applicable

Compared with all scenarios included in this analysis, Doherty Scenario 3C generated the highest morbidity and mortality impact followed by Scenario 3B with base case DALY burden of 94,998 (95%CI: 88,630-102,559) and 89,958 (95%CI:83,925-97,088) respectively. Including the potential impact of permanent functional impairment, the total DALY Burden One was 197,534 (95%CI: 189,182-206,262) for Scenario 3C and 185,174 (95%CI: 177,383-193,402) for Scenario 3B. Doherty Scenarios 2C and 2D generate significantly lower DALY burden. The base case DALY burden for Scenarios 2C and 2D were 21,915 (95%CI:20,278-23,852) and 13,695(95%CI:12,646-14,921) respectively. Total DALY Burden One including the potential burden of permanent function impairment was estimated as 49,046 (95%CI:47,006-51,193) for 2C and 30,705(95%CI:29,409-32,081) for 2D. In all these scenarios, the total DALY burden is significantly higher in unvaccinated persons than in those vaccinated. (Additional file 4: Table 9).

### Sensitivity analysis

**Fig. 2:**
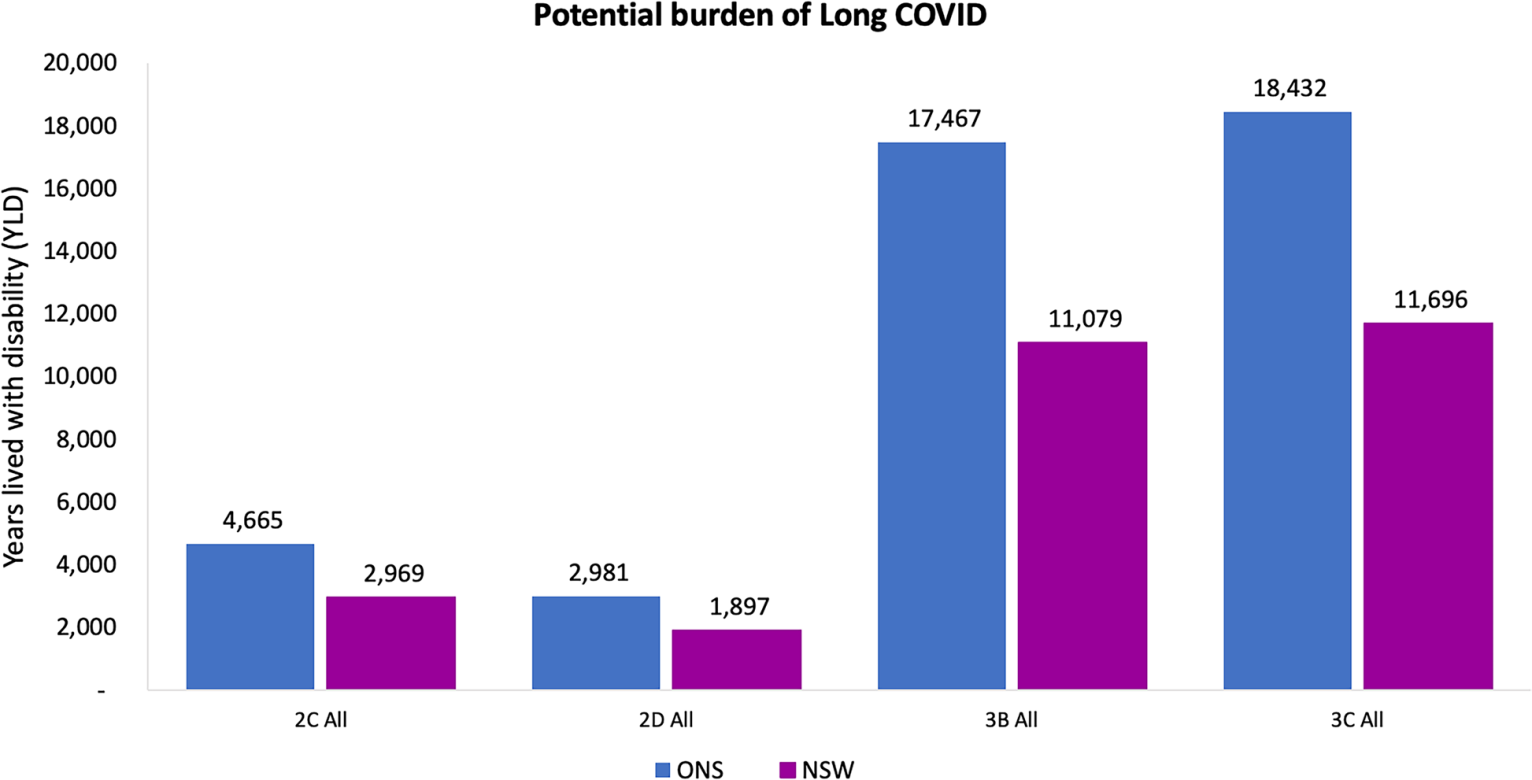
Potential Long COVID burden for all scenarios. Notes ONS = Office of the National Statistic data, NSW=New South Wales data

As expected, the burden for Long COVID decreased when the NSW estimate was used for Long COVID prevalence rather than ONS (Fig. 2), because the NSW-based prevalence estimates generates a lower estimate of YLD due to Long COVID than when the ONS-based estimate is used (Fig. 2 and Additional file 4: Table 9).

**Fig. 3:**
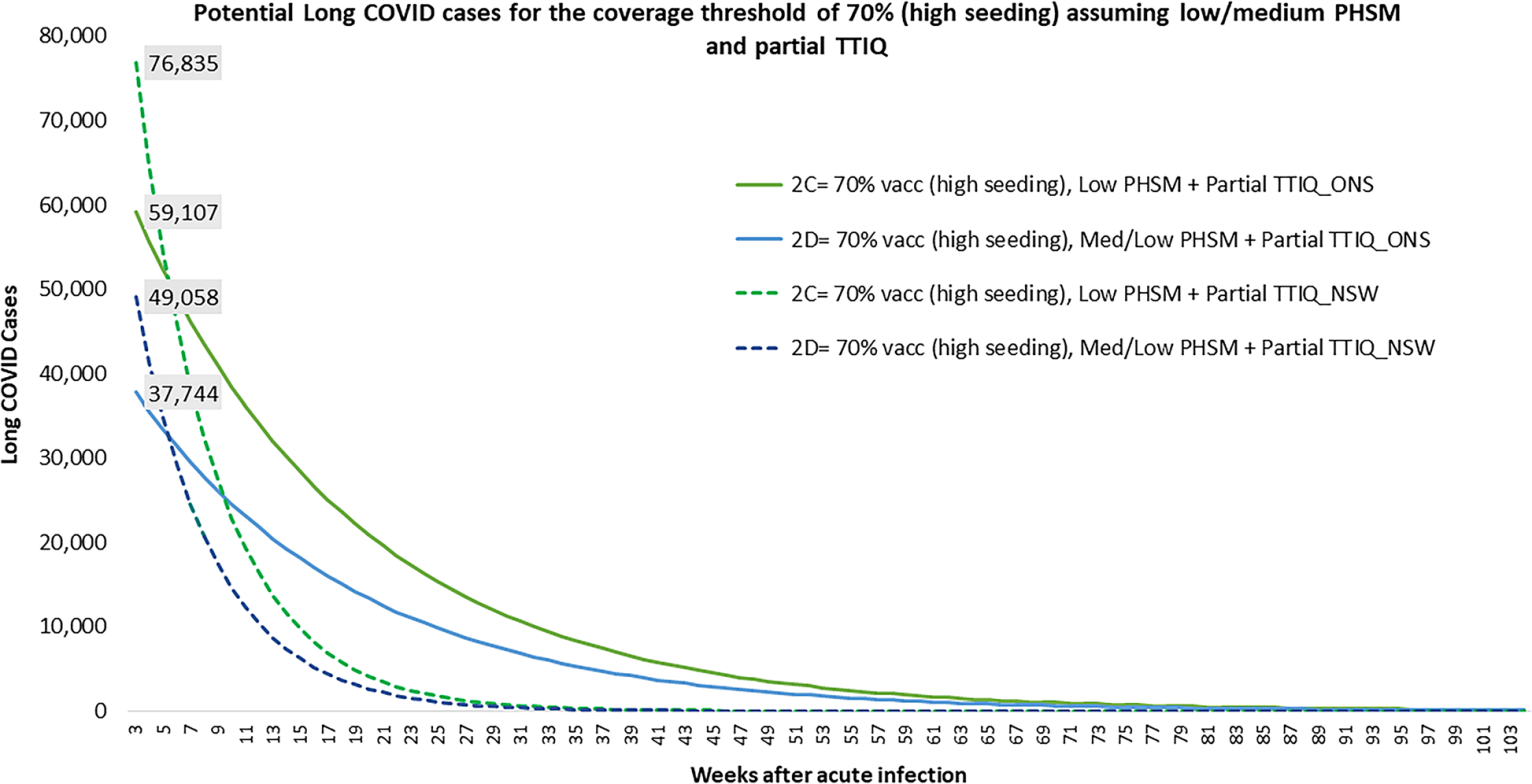
Potential Long COVID cases for Scenarios 2C and 2D. Notes: Solid lines presents the results using the ONS data points and Dashed lines presents the results using the NSW datapoints, results noted here excludes the impact of permanent disability and post-intensive care syndrome

**Fig. 4:**
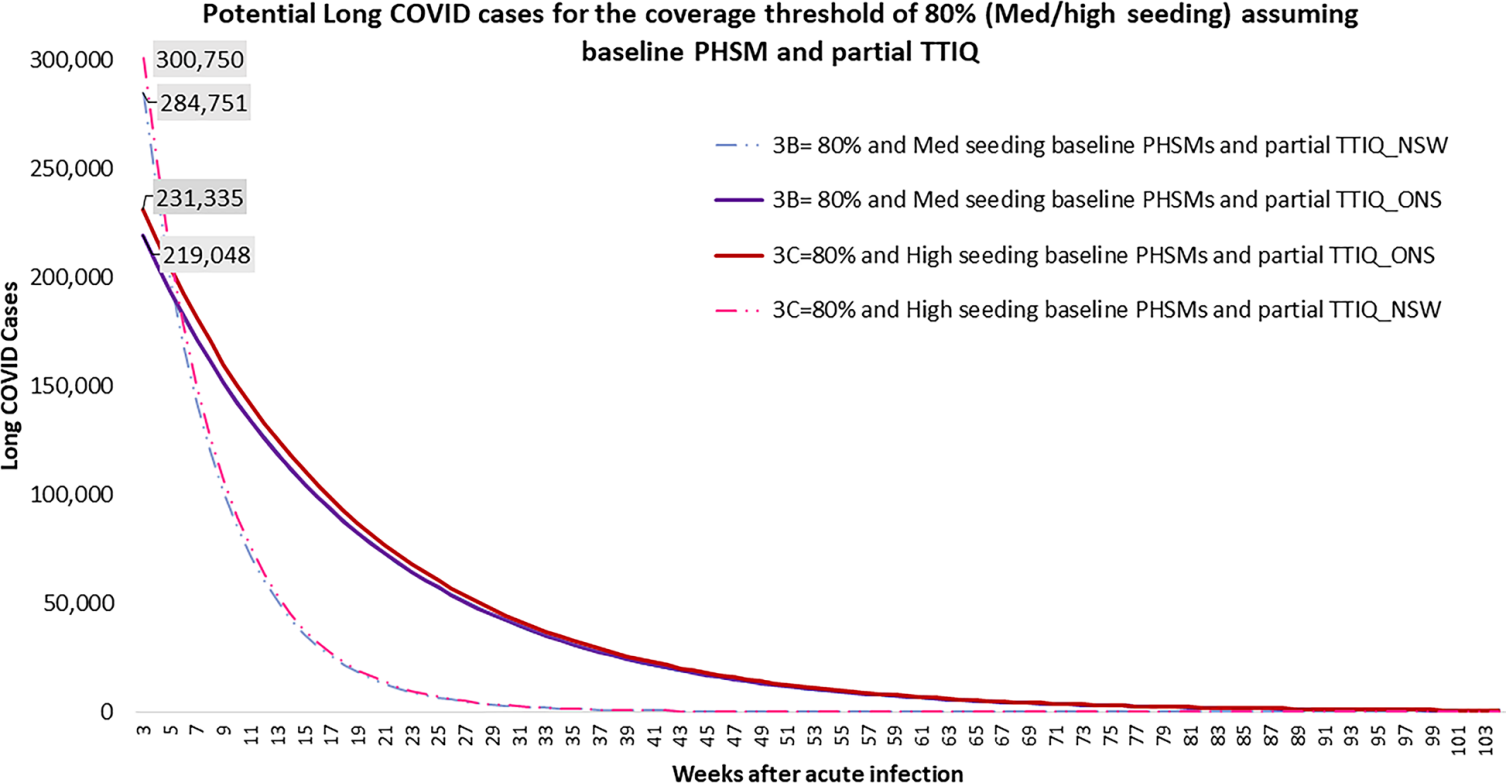
Potential Long COVID cases for Scenarios 3B and 3C. Notes: Solid lines presents the results using the ONS data points and Dashed lines presents the results using the NSW datapoints, results noted here excludes the impact of permanent disability and post-intensive care syndrome

Figures 3 and 4 show the potential number of Long COVID cases under Doherty Scenarios 2C-2D and 3B-3C respectively, using the ONS and NSW Long COVID data points. Scenario 2C and 2D (Fig. 3) represent outcomes where Australia has achieved a 70% vaccination coverage target observing a high seeding infection rate with partial TTIQ. In Scenario 2C, approximately 60,000 (ONS result) or 77,000 (NSW result) Long COVID cases are initially expected from the 244,875 modelled symptomatic COVID-19 infection assuming “low” PHSM. In contrast, a much lower number of Long COVID cases, at approximately 38,000 (ONS result) or 49,000 (NSW result) was estimated for Scenario 2D generated from the 155,851 modelled symptomatic COVID-19 cases assuming medium/low PHSM. For a given vaccine coverage threshold of 80% and assuming only “baseline” PHSM and partial TTIQ (Fig. 4– Scenarios 3B and 3C), both for medium and high seeding infection rate, more than 200,000 (ONS) or > 280,000 initial Long COVID cases were estimated from the Doherty model’s 908,000 to 961,000 COVID-19 cases. Both figures show how Long COVID case numbers reduce over time as patients recover. Notably, when NSW estimates were used in each scenario, higher Long COVID cases were initially observed, but Long COVID case numbers then declined quite rapidly. When ONS estimates were used, initial Long COVID case numbers are lower, but decline much more slowly.

## Discussion

To the best of our knowledge, this is the first study to illustrate the potential post-acute DALY losses arising from the hypothetical scenarios used by the Doherty Institute [6] to inform Australia’s roadmap to re-opening its international borders. Based on the Doherty modelling, our study found that even with 80% full dose vaccination coverage, up to 230,000 initial Long COVID cases might be expected if Australia re-opens at a medium or high seeding infection rate (partial TTIQ) with only baseline PHSMs. However, if the Australian vaccination target is at least 70% *and* low or medium PHSM are implemented, Long COVID cases will be much lower, even if there is a high seeding infection (partial TTIQ) rate upon re-opening.

The Doherty modelling accounts for the impacts of vaccination (and improved treatments) in reducing mortality from COVID-19. Our results show how this is reflected in a declining share of disease burden due to mortality compared with that seen in 2020 and how post-acute morbidity will drive an increasing share of COVID-19 disease burden in the future. Our study found that Long COVID was responsible for a large proportion of total YLDs (74-79%) in our base case DALY burden and accounted for 19-22% of the total DALYs lost across scenarios.

A key advance in our study was to consider the possible impact of the potential burden of permanent impairment due to COVID-19, which contributed up to 80-82% of the total YLD or 51-55% of potential total DALY losses in our model. Thus, this is the first study that have estimated the burden of post-acute sequelae of COVID-19 by DALYs. Earlier models had only calculated the impact of the acute phase of COVID-19 and of Long COVID [17, 42–46]. We found no other studies that had yet estimated the burden of PICS and the potentially significant burden of permanent disability in terms of DALYs. Our findings highlight that the impact of COVID-19 cannot be accurately assessed purely based on case numbers and deaths, but instead requires proper consideration of morbidity due to post-acute-COVID sequelae.

Our results emphasise the continuing importance of high levels of COVID-19 vaccination, not only to reduce mortality and severe acute illness but also to reduce the burden of Long COVID. At higher levels of vaccine coverage, the relative burden of longer-term morbidity increases, while that of mortality and acute illness decreases; healthcare system responses will increasingly need to deal with a larger post-acute burden of disease. Our results further emphasise a significant finding of the Doherty modelling – namely that, even with high vaccine coverage, specific choices of public health and social distancing measures still have significant impacts on case numbers. Overall COVID burden (acute and post-acute) is significantly higher under “baseline” (i.e. minimal) PHSMs than under low to medium settings. Data on the likely impacts on longer-term disease burden must therefore inform public health decision-making.

Our results indicate that Australian health systems need to prepare to care for thousands of patients with Long COVID or PICS over the coming months. As discussed elsewhere [7], this requires not only specialised Long COVID clinics but, just as importantly, good primary care support and care coordination in the community. Effective surveillance systems need to be established now to identify patients who may go on to develop longer-term sequelae and impairments in the months and years ahead.

We have used the Doherty modelling [6] where scaled values accounted for the transmission of Delta variant as our starting point for COVID-19 caseload. Our model, therefore, shares any limitations inherent to the original Doherty model, including its time horizon of 180 days for new infections. We note that asymptomatic cases were not reported in their report and therefore were excluded in this analysis. This might lead to an underestimate in our DALY estimates if post-COVID sequelae occur in some initially asymptomatic cases. As with similar simulation models, our model used many assumptions. The model only captured the age variation in calculation of YLL. Gender was not accounted for in this analysis given the limitations of the data available for use in our model. The number of cases and deaths presented in our primary source (Doherty modelling report) [6] were not stratified in a way that would allow us to account for gender appropriately. However, we have attempted to account for the impact of age in the calculation of YLL by multiplying the total number of deaths reported in the Doherty modelled result by the Mortality distribution rate obtained from the Australian National Interoperable Notifiable Disease Surveillance system dated 03 October 2021 [16]. The reason for selecting this method is that we assumed that the percentage distribution of cases for each age cohort in the actual Australian mortality data and hypothetical cases are similar. The disability weight for PICS was assumed to be equivalent to the QALY utility score of ICU COVID-19 survivors mapped to a similar illness with the same utility score and disability weights. The recovery duration of Long COVID is still unknown and therefore extrapolation of data was necessary. At the time of writing, 81% of people aged 16 and over had received two doses of vaccine, and Australia looks likely to achieve 90% coverage levels within weeks; higher vaccination rates will likely further reduce mortality and case numbers but were not included in the Doherty Institute modelling which we used for our estimates.

Knowledge of post-COVID-19 sequelae is still evolving, and there are inevitable uncertainties around the estimates used in the model. Only the most cited permanent disabilities from COVID-19 with reported incidence rate was included to illustrate the potential additional morbidity from the impact of permanent functional impairment. Thus, the model might underestimate the burden of permanent functional impairment following COVID-19 infection given that only selected diseases were included based on the existing evidence. Also, international literature was used to inform the scale of permanent disability due to the scarcity of Australian data.

The precise proportion of COVID-19 survivors who will experience Long COVID is still unknown and different studies reported a wide range of point estimates [7]. We used the UK ONS data [18], which is the largest, comprehensive and most robust study of Long COVID to date following a large population of confirmed COVID-19 infection (21,622). However, we employed the lower NSW population-based estimate [19] for sensitivity analysis resulted in a lower YLD burden of Long COVID that was only 63-64% of the burden of Long COVID estimated under the ONS base case across scenarios. Future research with more accurate data is needed to confirm the burden of Long COVID in Australia.

We undertook uncertainty analysis to account for the unavoidable uncertainty in the total DALY estimation. Key inputs such as symptomatic recovery duration were tested using the COVID-19 2020 cases, and only a small variation was observed to total DALYs.

This model assesses the likely magnitude of the overall burden of COVID-19 that can inform Australian and other policy-makers to prepare healthcare services and systems for the likely negative impacts beyond the acute phase of COVID-19 for Long COVID, PICS and permanent disability. A future COVID-19 burden of disease model could employ these health states to provide the likely impact of COVID-19 that goes beyond the acute phase of the pandemic.

## Conclusions

Our results emphasise that ongoing decisions on pandemic control measures and re-opening strategies need to incorporate more than simply numbers of expected infections and deaths. The burden of post-acute COVID-19 was already non-negligible during 2020. However, it becomes relatively more important as deaths fall with improved vaccination coverage. Emerging evidence of the potential for COVID survivors to develop significant long-term illness and disability – not to mention the impacts of Long COVID – means that policymakers in Australia and overseas will increasingly need to prepare for greater long-term demands for care and support. Our model provides a useful starting point for quantifying the burden involved, and can assist both planning and prioritisation efforts.

In particular, we have demonstrated that even low rates of incidence of COVID-related permanent illness or disability could still lead to a very significant future burden of disease. Investing now in effective surveillance systems to understand the real incidence and burden of Long COVID and permanent illness or impairment after COVID is therefore essential, in Australia and other jurisdictions. Even more urgently, health services must act rapidly to meet the emerging needs of patients with Long COVID, PICS and longer-term illness, not only in the present Victorian and New South Wales outbreaks, but also for the new cases likely to accompany the progressive re-opening of Australia as envisaged in the National Plan [6]. Moreover, future studies could further develop the existing COVID-19 burden model in light of the rapidly emerging and evolving nature of COVID-19, particularly with the knowledge and data availability on post-COVID-19 sequelae. Research priorities in this area will include systematic synthesis of emerging estimates of the incidence of permanent post-COVID illness and disability; healthcare costs and outcomes for people presenting with Long COVID and/or permanent post-COVID impairment; and the wider social and economic consequences of post-COVID sequelae.

## Supplementary Information

**Additional file 1.**

**Additional file 2.**

**Additional file 3.**

**Additional file 4.**

## Data Availability

All data generated or analysed during this study are included in this published article and provided in Additional files 1, 2, 3, and 4.

## Abbreviations

AIHW: Australian Institute of Health and Welfare
COVID-19: Coronavirus Disease 2019
DALYs: Disability adjusted life years
ICU: Intensive care unit
NSW: New South Wales
ONS: Office of National Statistics
OR: Odds ratio
PHSM: Public health and social measures
PICS: Post-intensive care syndrome
UK: United Kingdom
TTIQ: Efficacy of test, trace, isolate, quarantine
YLD: Years lived with disability
YLL: Years lost due to premature mortality
