# Supplementary material for: Modelling the potential acute and post-acute burden of COVID-19 under the Australian border re-opening plan
Source: BMC Public Health. 2022 Apr 14;22:757. doi: 10.1186/s12889-022-13169-x (PMC9009167; doi:10.1186/s12889-022-13169-x)
Supplement: Supplementary file 2 — Additional file 2. [file 12889_2022_13169_MOESM2_ESM.pdf]

Additional file 2

Additional parameters and information related to the inputs used in the Model

Table 1. All scenarios included in our study which were adapted based on Doherty's modelled COVID-19 outcomes

Scenarios	Description
**Scenario 1**	**70% and low seeding infections**
• Scenario 1A	Baseline PHSMs + Partial TTIQ effectiveness
• Scenario 1B	Baseline PHSMs + Optimal TTIQ effectiveness
• Scenario 1C	Low PHSMs + Partial TTIQ effectiveness
**Scenario 2**	**70% and high seeding infections**
• Scenario 2A	Baseline PHSM+ Partial TTIQ,
• Scenario 2B	Baseline PHSM+ Optimal TTIQ,
• Scenario 2C	Low PHSM+ Partial TTIQ,
• Scenario 2D	Med/Low + Partial§ TTIQ,
**Scenario 3**	**80% assuming baseline PHSMs and partial TTIQ**
• Scenario 3A	Low seeding infections
• Scenario 3B	Medium seeding infections
• Scenario 3C	High seeding infections
**Scenario 4**	**80% assuming baseline PHSMs and optimal TTIQ**
• Scenario 4A	Low seeding infections
• Scenario 4B	Medium seeding infections
• Scenario 4C	High seeding infections

Note: PHSM= public health and social measures, TTIQ= efficacy of test, trace, isolate, quarantine

Source: COVID-19 related deaths, symptomatic infections, ICU admission and ward admissions were reported in tables ES1, ES2, tables 2.3, and table 2.4 of Doherty's Modelling interim report to national cabinet 17th September 2021 [1].

Post-acute consequences

COVID-19 survivors were generated by deducting the total number of deaths from the total number of symptomatic infections. Doherty Modelling results reported the total number of patients admitted in the ICU and hospital ward. To get the number of ICU survivors, we applied the probability of dying from ICU[2] to patient's admitted in critical care. For the ward survivors, we directly deducted the ICU related deaths from the total deaths (ward related death) and deduct the total ward numbers from this estimate. Vaccinated individuals were less likely to have Long COVID and therefore a treatment effect of vaccines was applied in this cohort (Odds Ratio=0.51, 95%CI:0.32-0.82 converted to Relative risk). To correctly apply this estimate to our Long COVID health state, we then first convert our Long COVID probability to rates and then multiply RR and then convert it back probability [3-5].

As for the Long COVID probability we have used ONS and NSW datapoints and extrapolated it to 104 weeks. We have used the constant and power term in table 2 which were generated from the plotted data points of COVID Infection Survey and NSW population-based study. The data points for ONS and NSW were published elsewhere [6, 7]. Figures 1 and 2 present the plotted data points using a decay function similar to the methods of Martin et al [8].

Table 2. Data used to extrapolate the % post-acute consequences

CIS data points	Constant	Power term	Extrapolated data at 52 weeks	Extrapolated data at 104 weeks
Baseline	0.3111	-0.061	1.30%	0.1%
LL	0.2887	-0.073	0.60%	0.0%
UL	0.3358	-0.049	2.60%	0.2%
**NSW Data points**	**Constant**	**Power term**	**Extrapolated data at 52 weeks**	**Extrapolated data at 104 weeks**
Baseline	0.5629	-0.172	0.01%	0.00%
LL	0.5997	-0.199	0.00%	0.00%
UL	0.5276	-0.146	0.03%	0.00%

Note: CIS= COVID Infection Survey, NSW= New South Wales

Figure 1. Decay Curve for Long COVID using ONS data

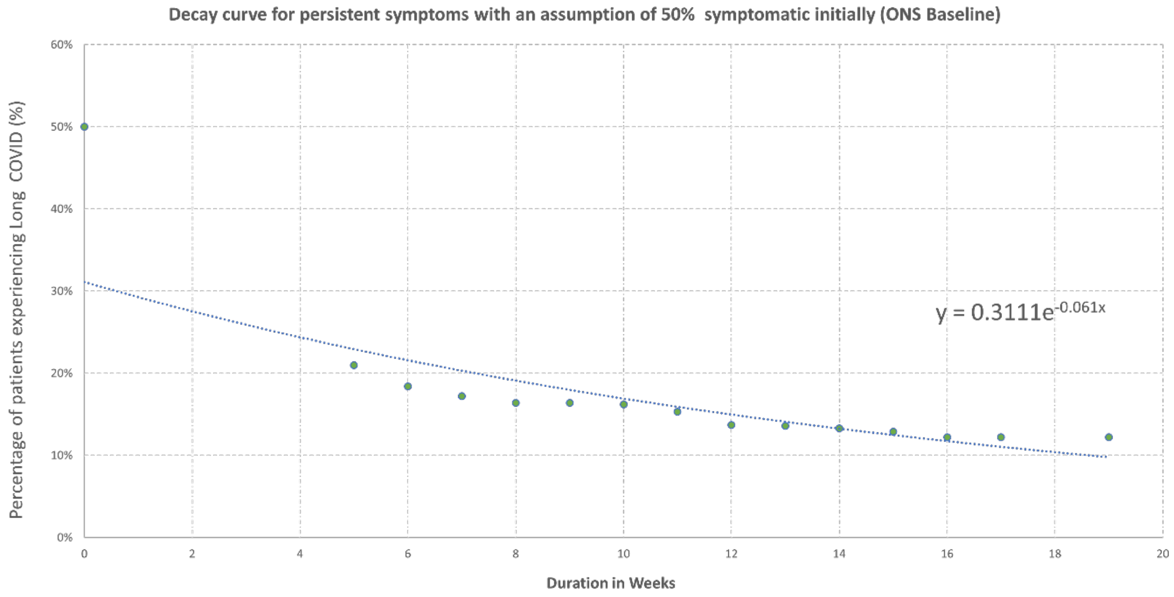

Source: Data sourced from the Office for National Statistics [7]

Figure 2: Decay Curve for Long COVID using NSW data

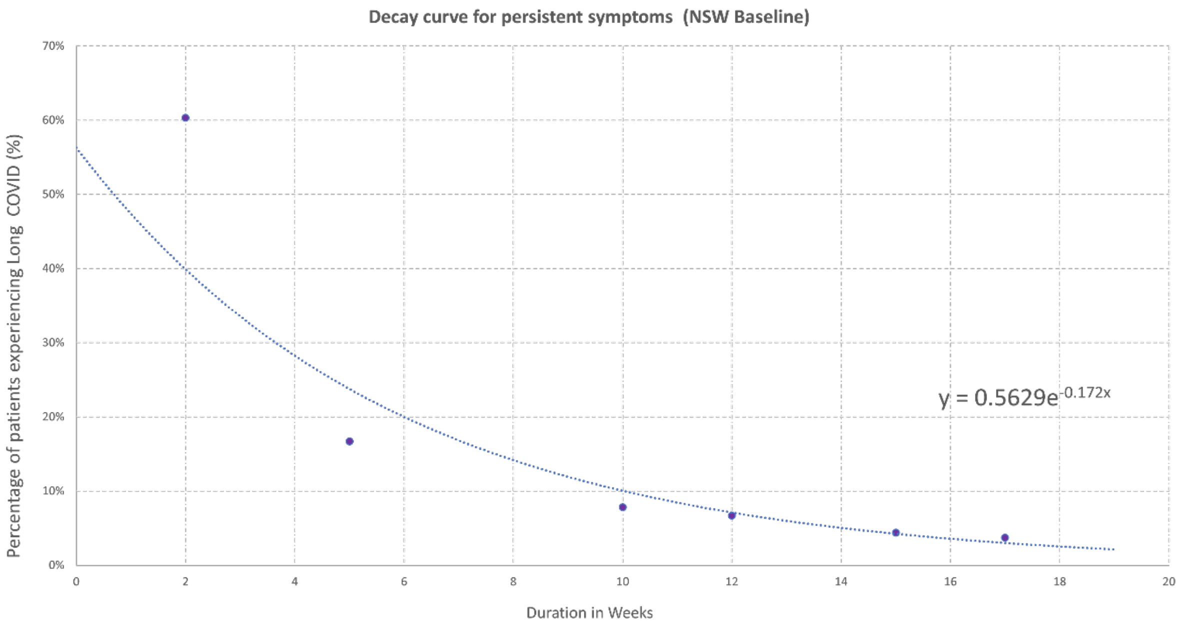

Source: Data sourced from Liu et al[6]

Post-Intensive Care Syndrome (PICS)

COVID-19 survivors aged 60 and above were considered in this cohort as the characteristics of patients developing PICS included in our chosen literature were in the age group of 65 (54-71) where more than half of the cohort are >65 years and 43.8% were retired [9]. ICU survival distribution per age group was obtained from Australia's Epidemiological report 50 [10].

References

1. Doherty Institute. Doherty Modelling Interim report to National Cabinet 17th September 2021 Australia: Doherty Institute; 2021; Cited 2021 September 20. Available from: https://www.doherty.edu.au/uploads/content_doc/DOHERTY_MODELLING_INTERIM_REPORT_TO_NATIONAL_CABINET_17TH_SEPTEMBER_2021.pdf.
2. Knock ES, Whittles LK, Lees JA, Perez-Guzman PN, Verity R, FitzJohn RG, et al. Key epidemiological drivers and impact of interventions in the 2020 SARS-CoV-2 epidemic in England. Sci Transl Med. 2021;13(602).
3. Antonelli M, Penfold RS, Merino J, Sudre CH, Molteni E, Berry S, et al. Risk factors and disease profile of post-vaccination SARS-CoV-2 infection in UK users of the COVID Symptom Study app: a prospective, community-based, nested, case-control study. The Lancet Infectious Diseases.
4. Zhang J, Yu KF. What's the Relative Risk?A Method of Correcting the Odds Ratio in Cohort Studies of Common Outcomes. JAMA. 1998;280(19):1690-1.
5. Fleurence RL, Hollenbeak CS. Rates and Probabilities in Economic Modelling. PharmacoEconomics. 2007;25(1):3-6.
6. Liu B, Jayasundara D, Pye V, Dobbins T, Dore GJ, Matthews G, et al. Whole of population-based cohort study of recovery time from COVID-19 in New South Wales Australia. The Lancet Regional Health-Western Pacific. 2021;12:100193.
7. Office for National Statistics. Prevalence of ongoing symptoms following coronavirus (COVID-19) infection in the UK: 1 April 2021: Estimates of the prevalence of self-reported "long COVID", and the duration of ongoing symptoms following confirmed coronavirus infection, using UK Coronavirus (COVID-19) Infection Survey data to 6 March 2021. UK: ONS; 2021; Cited 2021 September 01. Available from: https://www.ons.gov.uk/peoplepopulationandcommunity/healthandsocialcare/conditionsanddiseases/bulletins/prevalenceofongoingsymptomsfollowingcoronaviruscovid19infectionintheuk/1april2021.
8. Martin C, Luteijn M, Letton W, Robertson J, McDonald S. A model framework for projecting the prevalence and impact of Long-COVID in the UK. PLOS ONE. 2021;16(12):e0260843.
9. Rousseau A-F, Minguet P, Colson C, Kellens I, Chaabane S, Delanaye P, et al. Post-intensive care syndrome after a critical COVID-19: cohort study from a Belgian follow-up clinic. Annals of intensive care. 2021;11(1):1-9.
10. COVID-19 National Incident Room Surveillance Team. COVID-19 Australia: Epidemiology Report 50 reporting period ending 12 September 2021 2021; Cited 2021 October 01. Available from: https://www1.health.gov.au/internet/main/publishing.nsf/Content/C50CAE02452A48A7CA2587320081F7BF/$File/covid_19_australia_epidemiology_report_50_reporting_period_ending_12_september_2021.pdfsib.
