# Supplementary material for: Modelling the potential acute and post-acute burden of COVID-19 under the Australian border re-opening plan
Source: BMC Public Health. 2022 Apr 14;22:757. doi: 10.1186/s12889-022-13169-x (PMC9009167; doi:10.1186/s12889-022-13169-x)
Supplement: Supplementary file 3 — Additional file 3. [file 12889_2022_13169_MOESM3_ESM.pdf]

Additional file 3. Model Test inputs using the actual COVID-19 cases for 2020. This file shows the inputs and results used for the DALY model test

Table 1. Model tests input using the Actual COVID-19 cases from January 2020- January 2021

Health States	Rates (Sensitivity analysis)	Duration (Sensitivity Analysis)	DW (Uncertainty Analysis)
Asymptomatic	17% of the moderate cases[1]	Nil	Nil
Moderate	71.0% (Increased to 5% and 10%) to the whole Australian COVID-19 case[2, 3]	14 days (28 days)[4-6]	0.051 (0.032 – 0.074)*[7, 8]
Severe	10.1% (Increased to 5% and 10%) to the whole Australian COVID-19 case[2, 3]	14 days (28 days)[4-6]	0.133 (0.088 – 0.190)*[7, 8]
Critical	1.9% (Increased to 5% and 10%) to the whole Australian COVID-19 case[2, 3]	14 days (28 days)[4-6]	0.655 (0.579 – 0.727)*[7, 8]
Post-acute consequences (ONS)	Start at 25.91% (23.2% to 29.0%) 2 weeks after initial COVID infection[9]	14 days to 2 years (28 days to 2 years) (assumed)	0.219 (0.148-0.308)[7, 8]
Post-acute consequences (NSW)	Start at 33.60% (33.0% to 34.0%%) 2 weeks after initial COVID infection[10]	14 days to 2 years (28 days to 2 years) (assumed)	0.219 (0.148-0.308)[7, 8]
Post-Intensive Care Syndrome (PICS)	90.6% of ICU survivors[11]	14 days (28 days) to remaining lifetime expectancy[12, 13]	0.224 (0.151-0.312)* (a)[14, 15]
Permanent disability health states	Same as additional file 2 Table 4		

Table 2. Actual COVID-19 cases

Actual COVID-19 reported cases between January 2020 to 17 January 2021					
Age Group	Cumulative Cases				
	Cases			Number of Death	Cases without death
	Male	Female	People	People	People
0 to 9	792	711	1,503	0	1,503
10 to 19	1,254	1,197	2,451	0	2,451
20 to 29	3,067	3,366	6,455	1	6,454
30 to 39	2,587	2,493	5,095	2	5,093
40 to 49	1,890	1,788	3,706	2	3,704
50 to 59	1,647	1,741	3,396	15	3,381
60 to 69	1,199	1,220	2,421	38	2,383
70 to 79	856	756	1,612	157	1,455
80 to 89	495	780	1,275	379	896
90 and over	229	552	782	315	467
All	14,016	14,604	28,696	909	27,787

Source: COVID-19 Australia: Epidemiology Report 33 Fortnightly reporting period ending 17 January 202 and Department of Health COVID-19 case number and statistics 1[2, 16]

Model Test Results

Briefly, from January 2020 to January 2021, there were 28,696 cases and 909 deaths due to COVID-19 in Australia [2]. During this period, total base case burden of 8,855 (95%CI: 8,471-9,267) was estimated. Including the permanent functional impairment, a potential 12,467(95%CI: 12,039-12,903) was modelled arising from the 2020 COVID-19 outbreak (see Table 3).

Varying acute illness by up to 5% and 10% increase from baseline, did not meaningfully affect the results, with a total DALY of 12,469 (95%CI: 12,027-12,914) and 12,470 (95%CI: 12,034-12,939) respectively (Tables 4 and 5). Varying estimates of post-acute burden using NSW data, led YLDs to decrease from 565 to 361 compared to the baseline, but with a relatively small impact on total DALYs (12,269 (95%CI: 11,867-12,687)). Table 7 shows the result of the model when assumed recovery duration in the symptomatic phase increased from 14 to 28 days, the symptomatic YLD increased from 69 (95%CI:52-88) to 137 (95%CI:105-176), with only a small impact on total DALYs (12,514 (95%CI: 12,080-12,968)) compared to baseline DALY loss.

Table 3. DALYS Baseline Results (Using ONS Long COVID data points)

**Variable**	**Mean**	**LCI 95%**	**HCI 95%**
**YLL**	7,263.49	*	*
Symptomatic	68.53	52.38	87.68
Post-acute	564.92	375.16	791.60
PICS	958.35	635.52	1,322.12
Permanent functional impairment with D	3,611.31	3,444.61	3,794.39
Permanent functional impairment WOD	3,400.56	3,233.60	3,582.53
TOTAL YLD Baseline	1,591.80	1,207.41	2,003.45
TOTAL YLD with Diabetes	5,203.12	4,775.39	5,639.72
TOTAL YLD without Diabetes	4,992.36	4,569.16	5,431.28
TOTAL DALYS Baseline	8,855.29	8,470.90	9,266.93
Total DALYs with Diabetes	12,466.60	12,038.88	12,903.21
Total DALYs without Diabetes	12,255.85	11,832.64	12,694.77

*Note: YLL=Years of life lost, YLD= years lived with disability, UI= Uncertainty intervals, LCI= lower confidence interval, HCI= higher confidence interval, *= not applicable, PICS= post-intensive care syndrome, DALYs= Disability-adjusted life years*

Table 4. Sensitivity analysis: Results when symptomatic cases were increased to 5%

Variable	Mean	LCI 95%	HCI 95%
**YLL**	7,263.49	*	*
Symptomatic	72.40	56.14	93.33
Post-acute	563.36	362.85	796.72
PICS	957.28	638.12	1,326.23
Permanent functional impairment with D	3,612.53	3,441.50	3,793.19
Permanent functional impairment WOD	3,401.69	3,230.35	3,579.31
TOTAL YLD Baseline	1,593.03	1,198.36	2,006.69
TOTAL YLD with Diabetes	5,205.57	4,763.92	5,650.71
TOTAL YLD without Diabetes	4,994.72	4,557.60	5,442.79
TOTAL DALYS Baseline	8,856.52	8,461.85	9,270.17
Total DALYs with Diabetes	12,469.05	12,027.40	12,914.19
Total DALYs without Diabetes	12,258.20	11,821.09	12,706.28

*Note: YLL=Years of life lost, YLD= years lived with disability, UI= Uncertainty intervals, LCI= lower confidence interval, HCI= higher confidence interval, *= not applicable, PICS= post-intensive care syndrome, DALYs= Disability-adjusted life years*

Table 5. Results when symptomatic cases were increased to 10%

Variable	Mean	LCI 95%	HCI 95%
**YLL**	7,263.49		
Symptomatic	75.31	58.21	96.05
Post-acute	564.35	368.01	789.48
PICS	955.36	623.78	1,324.47
Permanent functional impairment with D	3,611.92	3,434.42	3,784.60
Permanent functional impairment WOD	3,401.20	3,228.68	3,575.30
TOTAL YLD Baseline	1,595.03	1,204.86	2,025.52
TOTAL YLD with Diabetes	5,206.95	4,770.86	5,675.23
TOTAL YLD without Diabetes	4,996.23	4,562.09	5,457.33
TOTAL DALYS Baseline	8,858.51	8,468.35	9,289.00
Total DALYs with Diabetes	12,470.43	12,034.35	12,938.71
Total DALYs without Diabetes	12,259.71	11,825.58	12,720.82

*Note: YLL=Years of life lost, YLD= years lived with disability, UI= Uncertainty intervals, LCI= lower confidence interval, HCI= higher confidence interval, *= not applicable, PICS= post-intensive care syndrome, DALYs= Disability-adjusted life years*

Table 6. Results when NSW was used in the post-acute consequence health state

Variable	Mean	LCI 95%	HCI 95%
**YLL**	7,263.49		
Symptomatic	68.34	52.07	86.69
Post-acute	360.67	236.06	506.96
PICS	961.42	642.22	1,317.82
Permanent functional impairment with D	3,614.89	3,440.59	3,794.41
Permanent functional impairment WOD	3,403.66	3,231.03	3,583.29
TOTAL YLD Baseline	1,390.42	1,024.35	1,775.30
TOTAL YLD with Diabetes	5,005.31	4,603.13	5,423.67
TOTAL YLD without Diabetes	4,794.09	4,392.12	5,211.63
TOTAL DALYS Baseline	8,653.91	8,287.84	9,038.78
Total DALYs with Diabetes	12,268.80	11,866.61	12,687.15
Total DALYs without Diabetes	12,057.57	11,655.61	12,475.11

*Note: YLL=Years of life lost, YLD= years lived with disability, UI= Uncertainty intervals, LCI= lower confidence interval, HCI= higher confidence interval, *= not applicable, PICS= post-intensive care syndrome, DALYs= Disability-adjusted life years*

Table 7. Results when 28-day symptomatic recovery duration was used

Variable	Mean	LCI 95%	HCI 95%
**YLL**	7,263.49	*	*
Symptomatic	136.92	104.95	175.91
Post-acute	549.21	362.09	750.60
PICS	952.00	628.80	1,300.68
Permanent functional impairment with D	3,612.65	3,444.80	3,790.31
Permanent functional impairment WOD	3,401.62	3,235.69	3,575.68
TOTAL YLD Baseline	1,638.13	1,253.81	2,051.73
TOTAL YLD with Diabetes	5,250.78	4,816.26	5,704.92
TOTAL YLD without Diabetes	5,039.74	4,609.23	5,500.78
TOTAL DALYS Baseline	8,901.61	8,517.29	9,315.22
Total DALYs with Diabetes	12,514.26	12,079.74	12,968.40
Total DALYs without Diabetes	12,303.23	11,872.71	12,764.26

*Note: YLL=Years of life lost, YLD= years lived with disability, UI= Uncertainty intervals, LCI= lower confidence interval, HCI= higher confidence interval, *= not applicable, PICS= post-intensive care syndrome, DALYs= Disability-adjusted life years*

References

1. Byambasuren O, Cardona M, Bell K, Clark J, McLaws M-L, Glasziou P. Estimating the extent of asymptomatic COVID-19 and its potential for community transmission: systematic review and meta-analysis. Official Journal of the Association of Medical Microbiology and Infectious Disease Canada. 2020;5(4):223-34.
2. COVID-19 National Incident Room Surveillance Team. COVID-19 Australia: Epidemiology Report 33 Fortnightly reporting period ending 17 January 2021 2021; Cited 2021 September 01. Available from: https://www1.health.gov.au/internet/main/publishing.nsf/Content/novel_coronavirus_2019_ncov_weekly_epidemiology_reports_australia_2020.htm.
3. COVID-19 National Incident Room Surveillance Team. COVID-19 Australia: Epidemiology Report 42 Reporting period ending 23 May 2021. Commun Dis Intell (2018). 2021;45.
4. The National Institute for Health and Care Excellence (NICE). COVID-19 guideline scope: management of the long-term effects of COVID-19: The National Institute for Health and Care Excellence (NICE); 2020; Cited 2021 August 01. Available from: https://www.nice.org.uk/guidance/ng188/documents/final-scope.
5. Rajan S, Khunti K, Alwan N, Steves C, MacDermott N, Morsella A, et al. In the wake of the pandemic: preparing for Long COVID: WHO Regional Office for Europe; 2021; Cited 2021 August 01. Available from: https://www.euro.who.int/en/health-topics/health-emergencies/coronavirus-covid-19/publications-and-technical-guidance/2021/in-the-wake-of-the-pandemic-preparing-for-long-covid-2021.
6. Cuschieri S, Calleja N, Devleesschauwer B, Wyper GMA. Estimating the direct Covid-19 disability-adjusted life years impact on the Malta population for the first full year. BMC Public Health. 2021;21(1):1827.
7. European Burden of Disease Network. Burden of disease of COVID-19: Protocol for country studies 2020; Cited 2021 August 01 Available from: https://www.burden-eu.net/docs/covid19-bod-protocol.pdf.
8. Wyper G, Assunção R, Colzani E, Grant I, Haagsma JA, Lagerweij G, et al. Burden of disease methods: a guide to calculate COVID-19 disability-adjusted life years. International Journal of Public Health. 2021;66:4.
9. Office for National Statistics. Prevalence of ongoing symptoms following coronavirus (COVID-19) infection in the UK: 1 April 2021: Estimates of the prevalence of self-reported "long COVID", and the duration of ongoing symptoms following confirmed coronavirus infection, using UK Coronavirus (COVID-19) Infection Survey data to 6 March 2021. UK: ONS; 2021; Cited 2021 September 01. Available from: https://www.ons.gov.uk/peoplepopulationandcommunity/healthandsocialcare/conditionsanddiseases/bulletins/prevalenceofongoingsymptomsfollowingcoronaviruscovid19infectionintheuk/1april2021.
10. Liu B, Jayasundara D, Pye V, Dobbins T, Dore GJ, Matthews G, et al. Whole of population-based cohort study of recovery time from COVID-19 in New South Wales Australia. The Lancet Regional Health-Western Pacific. 2021;12:100193.
11. Rousseau A-F, Minguet P, Colson C, Kellens I, Chaabane S, Delanaye P, et al. Post-intensive care syndrome after a critical COVID-19: cohort study from a Belgian follow-up clinic. Annals of intensive care. 2021;11(1):1-9.
12. Hofhuis JG, Schrijvers AJ, Schermer T, Spronk PE. Health-related quality of life in ICU survivors—10 years later. Scientific Reports. 2021;11(1):1-10.

13. Cuthbertson BH, Roughton S, Jenkinson D, Maclennan G, Vale L. Quality of life in the five years after intensive care: a cohort study. Crit Care. 2010;14(1):R6-R.
14. Byrnes J, Ball J, Gao L, Kai Chan Y, Kularatna S, Stewart S, et al. Within trial cost-utility analysis of disease management program for patients hospitalized with atrial fibrillation: results from the SAFETY trial. Journal of medical economics. 2019;22(9):945-52.
15. Global Burden of Disease Collaborative Network. Global Burden of Disease Study 2019 (GBD 2019) Disability Weights Seattle, United States: Institute for Health Metrics and Evaluation (IHME); 2020; Cited 2021 August 01 Available from: http://ghdx.healthdata.org/record/ihme-data/gbd-2019-disability-weights.
16. Department of Health. Coronavirus (COVID-19) case numbers and statistics: Department of Health,; 2021; Cited 2021 June 22. Available from: https://www.health.gov.au/news/health-alerts/novel-coronavirus-2019-ncov-health-alert/coronavirus-covid-19-current-situation-and-case-numbers.
