# Supplementary material for: Modelling the potential acute and post-acute burden of COVID-19 under the Australian border re-opening plan
Source: BMC Public Health. 2022 Apr 14;22:757. doi: 10.1186/s12889-022-13169-x (PMC9009167; doi:10.1186/s12889-022-13169-x)
Supplement: Supplementary file 4 — Additional file 4. [file 12889_2022_13169_MOESM4_ESM.pdf]

Additional file 4. Full results of the model. This file provides full results of the DALY COVID-19 burden upon Australia's reopening.

Table 1. Results for Scenario 1 (Using ONS data points for Long COVID)

**Scenario 1**	**Scenario 1A**		**Scenario 1B**		**Scenario 1C**	
	**Mean**	**95%CI LL and UL**	**Mean**	**95%CI LL and UL**	**Mean**	**95%CI LL and UL**
**YLL**	59,462		1,107		439	
• Symptomatic	1,988	(1389-2716)	48	(33-67)	18	(12-25)
• Long COVID	16,656	(10909-23363)	423	(277-594)	155	(101-217)
• PICS	3,399	(2264-4619)	56	(37-76)	24	(16-32)
• Permanent functional impairment with Diabetes	90,449	(86544-94711)	2,151	(2058-2252)	824	(788-862)
• Permanent functional impairment without diabetes	70,164	(66633-73766)	1,692	(1607-1779)	646	(613-679)
TOTAL YLD Baseline	22,043	(16250-28857)	527	(381-700)	197	(143-260
TOTAL YLD with Diabetes	112,492	(105476-120235)	2,677	(2502-2868)	1,020	(955-1091)
TOTAL YLD without Diabetes	92,207	(85236-99744)	2,219	(2047-2404)	842	(778-911)
TOTAL DALYS Baseline	81,506	(75713-88320)	1,633	(1487-1807)	635	(581-699)
Total DALYs with Diabetes	171,955	(164938-179697)	3,784	(3609-3975)	1,459	(1394-1529)
Total DALYs without Diabetes	151,670	(144699-159206)	3,325	(3154-3511)	1,281	(1217-1349)

Note: YLL=Years of life lost, YLD= years lived with disability, LL= lower confidence interval, UL= higher confidence interval, PICS= post-intensive care syndrome, DALYs= Disability-adjusted life years, results are referring to the combined mean burden of both vaccinated and unvaccinated individuals

Table 2. Results for Scenario 2 (Using ONS data points for Long COVID)

Scenario 2	Scenario 2A		Scenario 2B		Scenario 2C		Scenario 2D	
	Mean	95%CI LL and UL	Mean	95%CI LL and UL	Mean	95%CI LL and UL	Mean	95%CI LL and UL
**YLL**	92,644		28,891		15,912		9,898	
• Symptomatic	2,783	(1959-3788)	1,001	(696-1370)	558	(388-765)	353	(245-485)
• Long COVID	22,325	(14862-31414)	8,337	(5546-11716)	4,665	(3102-6555)	2,981	(1982-4188)
• PICS	5,392	(3542-7506)	1,459	(959-2031)	779	(512-1084)	462	(304-643)
• Permanent functional impairment with Diabetes	135,954	(130120-142247)	48,240	(46173-50471)	27,131	(25968-28387)	17,009	(16281-17798)
• Permanent functional impairment without diabetes	104,685	(99460-110191)	37,509	(35637-39482)	21,113	(20059-22224)	13,263	(12601-13960)
TOTAL YLD Baseline	30,499	(22544-39997)	10,797	(7861-14281)	6,003	(4365-7939)	3,797	(2748-5023)
TOTAL YLD with Diabetes	166,453	(156365-177243)	59,037	(55399-62893)	33,134	(31094-35281)	20,807	(19511-22183)
TOTAL YLD without Diabetes	135,184	(126057-145806)	48,306	(44985-52161)	27,116	(25257-29267)	17,060	(15883-18426)
TOTAL DALYS Baseline	123,144	(115189-132641)	39,687	(36752-43171)	21,915	(20278-23852)	13,695	(12646-14921)
Total DALYs with Diabetes	259,097	(249010-269887)	87,928	(84289-91783)	49,046	(47006-51193)	30,705	(29409-32081)
Total DALYs without Diabetes	227,829	(218702-238451)	77,197	(73875-81052)	43,028	(41169-45179)	26,958	(25781-28324)

Note: YLL= Years of life lost, YLD= years lived with disability, LL= lower confidence interval, UL= higher confidence interval, PICS= post-intensive care syndrome, DALYs= Disability-adjusted life years, results are referring to the combined mean burden of both vaccinated and unvaccinated individuals

Table 3. Results for Scenario 3 (Using ONS data points for Long COVID)

Scenario 3	Scenario 3A		Scenario 3B		Scenario 3C	
	**Mean**	**95%CI LL and UL**	**Mean**	**95%CI LL and UL**	**Mean**	**95%CI LL and UL**
**YLL**	42,892		66,844		70,154	
• Symptomatic	1,643	(1113-2262)	2,069	(1422-2825)	2,213	(1532-3014)
• Long COVID	14,384	(9477-20040)	17,467	(11501-24384)	18,432	(12137-25742)
• PICS	2,006	(1319-2787)	3,578	(2352-4970)	4,198	(2760-5831)
• Permanent functional impairment with Diabetes	76,385	(72895-80020)	95,215	(90875-99751)	102,537	(97868-107421)
• Permanent functional impairment without diabetes	59,420	(56263-62619)	73,804	(69884-77779)	79,345	(75130-83617)
TOTAL YLD Baseline	18,033	(13135-23774)	23,114	(17081-30244)	24,843	(18476-32404)
TOTAL YLD with Diabetes	94,418	(88146-101227)	118,329	(110538-126558)	127,380	(119028-136108)
TOTA YLD without Diabetes	77,453	(71499-83869)	96,918	(89566-104768)	104,188	(96388-112481)
TOTAL DALYS Baseline	60,926	(56027-66667)	89,958	(83925-97088)	94,998	(88630-102559)
Total DALYs with Diabetes	137,310	(131038-144120)	185,174	(177383-193402)	197,534	(189182-206262)
Total DALYs without Diabetes	120,345	(114391-126761)	163,763	(156411-171613)	174,343	(166543-182635)

Note: YLL= Years of life lost, YLD= years lived with disability, LL= lower confidence interval, UL= higher confidence interval, PICS= post-intensive care syndrome, DALYs= Disability-adjusted life years, results are referring to the combined mean burden of both vaccinated and unvaccinated individuals

Table 4. Results for Scenario 4 (Using ONS data points for Long COVID)

Scenario 4	Scenario 4A		Scenario 4B		Scenario 4C	
	**Mean**	**95%CI LL and UL**	**Mean**	**95%CI LL and UL**	**Mean**	**95%CI LL and UL**
**YLL**	282		3,905		17,520	
• Symptomatic	12	(8-17)	159	(106-220)	615	(416-845)
• Long COVID	109	(73-155)	1,416	(945-2009)	5,307	(3537-7533)
• PICS	13	(8-17)	155	(99-214)	781	(497-1075)
• Permanent functional impairment with Diabetes	545	(521-570)	7,215	(6897-7535)	28,434	(27181-29700)
• Permanent functional impairment without diabetes	430	(409-453)	5,676	(5392-5972)	22,236	(21125-23398)
TOTAL YLD Baseline	134	(98-180)	1,730	(1256-2323)	6,703	(4909-8918)
TOTAL YLD with Diabetes	679	(636-731)	8,946	(8376-9604)	35,137	(32943-37654)
TOTAL YLD without Diabetes	564	(522-614)	7,406	(6863-8050)	28,939	(26860-31338)
TOTAL DALYS Baseline	416	(379-462)	5,635	(5161-6228)	24,223	(22430-26438)
Total DALYs with Diabetes	961	(917-1012)	12,851	(12281-13509)	52,657	(50463-55174)
Total DALYs without Diabetes	846	(804-896)	11,311	(10768-11955)	46,459	(44380-48858)

Note: YLL= Years of life lost, YLD= years lived with disability, LL= lower confidence interval, UL= higher confidence interval, PICS= post-intensive care syndrome, DALYs= Disability-adjusted life years, results are referring to the combined mean burden of both vaccinated and unvaccinated individuals

Table 5. Results for Scenario 1 (Using NSW data points for Long COVID)

Scenario 1	Scenario 1A		Scenario 1B		Scenario 1C	
	**Mean**	**95%CI LL and UL**	**Mean**	**95%CI LL and UL**	**Mean**	**95%CI LL and UL**
**YLL**	59,462		28,891		439	
• Symptomatic	1,989	(1396-2736)	996	(688-1368)	18	(13-25)
• Long COVID	10,615	(6981-14954)	5,304	(3567-7365)	99	(65-139)
• PICS	3,410	(2296-4686)	1,458	(976-1993)	24	(16-33)
• Permanent functional impairment with Diabetes	90,492	(86424-94544)	48,194	(45951-50430)	824	(787-861)
• Permanent functional impairment without diabetes	70,172	(66533-73823)	37,473	(35483-39492)	646	(612-679)
TOTAL YLD Baseline	16,014	(12190-20598)	7,758	(5963-9972)	140	(106-183)
TOTAL YLD with Diabetes	106,505	(100694-112442)	55,952	(53013-59045)	964	(912-1019)
TOTAL YLD without Diabetes	86,186	(80717-91950)	45,231	(42525-48090)	786	(736-839)
TOTAL DALYS Baseline	75,476	(71652-80061)	36,649	(34854-38863)	579	(545-621)
Total DALYs with Diabetes	165,968	(160156-171904)	84,842	(81904-87936)	1,403	(1350-1457)
Total DALYs without Diabetes	145,648	(140179-151413)	74,122	(71416-76980)	1,225	(1174-1277)

Note: YLL= Years of life lost, YLD= years lived with disability, LL= lower confidence interval, UL= higher confidence interval, PICS= post-intensive care syndrome, DALYs= Disability-adjusted life years, results are referring to the combined mean burden of both vaccinated and unvaccinated individuals

Table 6. Results for Scenario 2 (Using NSW data points for Long COVID)

Scenario 2	Scenario 2A		Scenario 2B		Scenario 2C		Scenario 2D	
	**Mean**	**95%CI LL and UL**	**Mean**	**95%CI LL and UL**	Mean	**95%CI LL and UL**	**Mean**	**95%CI LL and UL**
**YLL**	92,644		28,891		15,912		9,898	
• Symptomatic	2,769	(1940-3780)	996	(688-1368)	555	(384-764)	352	(242-484)
• Long COVID	14,214	(9581-19756)	5,304	(3567-7365)	2,969	(1996-4123)	1,897	(1274-2634)
• PICS	5,390	(3608-7366)	1,458	(976-1993)	779	(521-1064)	462	(309-631)
• Permanent functional impairment with Diabetes	135,822	(129488-142116)	48,194	(45951-50430)	27,105	(25844-28362)	16,993	(16204-17780)
• Permanent functional impairment without diabetes	104,583	(99030-110221)	37,473	(35483-39493)	21,093	(19973-22230)	13,250	(12546-13964)
TOTAL YLD Baseline	22,373	(17380-28461)	7,758	(5963-9972)	4,303	(3307-5541)	2,710	(2080-3501)
TOTAL YLD with Diabetes	158,194	(150032-166796)	55,952	(53013-59045)	31,408	(29761-33135)	19,703	(18667-20798)
TOTAL YLD without Diabetes	126,956	(119665-134883)	45,231	(42525-48090)	25,396	(23878-27008)	15,960	(14998-16975)
TOTAL DALYS Baseline	115,017	(110025-121106)	36,649	(34854-38863)	20,216	(19219-21454)	12,608	(11978-13399)
Total DALYs with Diabetes	250,839	(242677-259440)	84,842	(81904-87936)	47,320	(45673-49047)	29,601	(28566-30696)
Total DALYs without Diabetes	219,600	(212309-227527)	74,122	(71416-76980)	41,308	(39791-42921)	25,858	(24896-26874)

Note: YLL= Years of life lost, YLD= years lived with disability, LL= lower confidence interval, UL= higher confidence interval, PICS= post-intensive care syndrome, DALYs= Disability-adjusted life years, results are referring to the combined mean burden of both vaccinated and unvaccinated individuals

Table 7. Results for Scenario 3 (Using NSW data points for Long COVID)

Scenario 3	Scenario 3A		Scenario 3B		Scenario 3C	
	**Mean**	**95%CI LL and UL**	**Mean**	**95%CI LL and UL**	**Mean**	**95%CI LL and UL**
**YLL**	42,892		66,844		70,154	
• Symptomatic	1,645	(1117-2289)	2,071	(1430-2854)	2,216	(1539-3044)
• Long COVID	9,119	(6159-12651)	11,079	(7487-15367)	11,696	(7906-16233)
• PICS	2,008	(1352-2763)	3,580	(2411-4926)	4,201	(2829-5781)
• Permanent functional impairment with Diabetes	76,416	(73037-80017)	95,254	(91028-99766)	102,579	(98001-107435)
• Permanent functional impairment without diabetes	59,444	(56590-62610)	73,834	(70290-77766)	79,377	(75566-83605)
TOTAL YLD Baseline	12,772	(9603-16402)	16,730	(12807-21185)	18,112	(13875-22899)
TOTAL YLD with Diabetes	89,188	(84363-93985)	111,984	(105940-118036)	120,691	(114186-127166)
TOTAL YLD without Diabetes	72,215	(67784-76807)	90,564	(85087-96291)	97,489	(91618-103661)
TOTAL DALYS Baseline	55,664	(52495-59295)	83,575	(79652-88029)	88,267	(84029-93053)
Total DALYs with Diabetes	132,080	(127255-136878)	178,829	(172785-184880)	190,845	(184340-197320)
Total DALYs without Diabetes	115,108	(110677-119699)	157,409	(151931-163135)	167,643	(161772-173815)

Note: YLL= Years of life lost, YLD= years lived with disability, LL= lower confidence interval, UL= higher confidence interval, PICS= post-intensive care syndrome, DALYs= Disability-adjusted life years, results are referring to the combined mean burden of both vaccinated and unvaccinated individuals

Table 8. Results for Scenario 4 (Using NSW data points for Long COVID)

Scenario 4	Scenario 4A		Scenario 4B		Scenario 4C	
	**Mean**	**95%CI LL and UL**	**Mean**	**95%CI LL and UL**	**Mean**	**95%CI LL and UL**
**YLL**	282		3,905		17,520	
• Symptomatic	12	(8-17)	159	(106-226)	614	(417-870)
• Long COVID	69	(46-96)	895	(600-1239)	3,359	(2251-4647)
• PICS	12	(8-17)	154	(104-214)	773	(525-1078)
• Permanent functional impairment with Diabetes	545	(521-571)	7,213	(6892-7553)	28,427	(27164-29769)
• Permanent functional impairment without diabetes	430	(409-452)	5,676	(5396-5969)	22,238	(21141-23384)
TOTAL YLD Baseline	94	(70-120)	1,208	(902-1556)	4,746	(3582-6071)
TOTAL YLD with Diabetes	639	(604-675)	8,421	(7967-8902)	33,173	(31444-35020)
TOTAL YLD without Diabetes	524	(491-558)	6,884	(6452-7339)	26,984	(25335-28719)
TOTAL DALYS Baseline	376	(352-402)	5,113	(4807-5461)	22,266	(21102-23591)
Total DALYs with Diabetes	921	(886-957)	12,326	(11872-12807)	50,693	(48965-52541)
Total DALYs without Diabetes	806	(773-840)	10,789	(10357-11244)	44,505	(42855-46239)

Note: YLL= Years of life lost, YLD= years lived with disability, LL= lower confidence interval, UL= higher confidence interval, PICS= post-intensive care syndrome, DALYs= Disability-adjusted life years, results are referring to the combined mean burden of both vaccinated and unvaccinated individuals

Table 9. Mean summarized results for all scenarios (ONS result)

Mean ONS												
**Variable**	**Vaccinated**				**Unvaccinated**				**Vaccinated and unvaccinated**			
	**2C Vacc'd**	**2D Vacc'd**	**3B Vacc'd**	**3C Vacc'd**	**2C Unvac**	**2D Unvac**	**3B Unvac**	**3C Unvac**	**2C All**	**2D All**	**3B All**	**3C All**
YLL	4,907	3,017	21,801	23,879	11,005	6,881	45,043	46,275	15,912	9,898	66,844	70,154
Symptomatic	99	60	380	421	459	293	1,689	1,792	558	353	2,069	2,213
Long COVID	433	263	1,638	1,796	4,233	2,719	15,830	16,636	4,665	2,981	17,467	18,432
PICS	371	219	1,700	2,005	408	243	1,878	2,193	779	462	3,578	4,198
Permanent functional impairment with diabetes	7,326	4,463	27,962	30,575	19,805	12,546	67,254	71,962	27,131	17,00	95,215	102,537
Permanent functional impairment Without diabetes	5,172	3,143	19,296	21,166	15,941	10,119	54,509	58,179	21,113	13,263	73,804	79,345
TOTAL YLD Baseline	903	542	3,717	4,223	5,099	3,255	19,396	20,621	6,003	3,797	23,114	24,843
TOTAL YLD with Diabetes	8,229	5,006	31,679	34,798	24,905	15,801	86,650	92,583	33,134	20,807	118,329	127,380
TOTAL YLD without Diabetes	6,075	3,685	23,013	25,389	21,040	13,374	73,905	78,799	27,116	17,060	96,918	104,188
TOTAL DALYS Baseline	5,810	3,560	25,519	28,102	16,104	10,136	64,440	66,896	21,915	13,695	89,958	94,998
Total DALYs with Diabetes	13,136	8,023	53,480	58,677	35,910	22,682	131,693	138,858	49,046	30,705	185,174	197,534
Total DALYs without Diabetes	10,983	6,703	44,814	49,268	32,045	20,255	118,948	125,075	43,028	26,958	163,763	174,343
**Mean NSW (Sensitivity Analysis)**												
**Variable**	**2C Vacc'd**	**2D Vacc'd**	**3B Vacc'd**	**3C Vacc'd**	**2C Unvac**	**2D Unvac**	**3B Unvac**	**3C Unvac**	**2C All**	**2D All**	**3B All**	**3C All**
YLL	4,907	3,017	21,801	23,879	11,005	6,881	45,043	46,275	15,912	9,898	66,844	70,154
Symptomatic	99	60	381	422	457	292	1,690	1,794	555	352	2,071	2,216
Long COVID	279	170	1,053	1,155	2,690	1,727	10,026	10,541	2,969	1,897	11,079	11,696
PICS	371	219	1,701	2,006	408	243	1,879	2,195	779	462	3,580	4,201
Permanent functional impairment with diabetes	7,319	4,459	27,973	30,587	19,786	12,534	67,281	71,991	27,105	16,993	95,254	102,579
Permanent functional impairment Without diabetes	5,167	3,140	19,303	21,175	15,925	10,109	54,531	58,202	21,093	13,250	73,834	79,377
TOTAL YLD Baseline	749	449	3,135	3,583	3,554	2,262	13,596	14,529	4,303	2,710	16,730	18,112

TOTAL YLD with Diabetes	8,068	4,908	31,108	34,171	23,340	14,796	80,877	86,520	31,408	19,703	111,984	120,691
TOTAL YLD without Diabetes	5,916	3,589	22,438	24,758	19,480	12,371	68,126	72,731	25,396	15,960	90,564	97,489
TOTAL DALYS Baseline	5,656	3,466	24,936	27,462	14,559	9,142	58,639	60,804	20,216	12,608	83,575	88,267
Total DALYs with Diabetes	12,975	7,925	52,909	58,050	34,345	21,676	125,920	132,796	47,320	29,601	178,829	190,845
Total DALYs without Diabetes	10,824	6,606	44,239	48,637	30,485	19,252	113,169	119,007	41,308	25,858	157,409	167,643

Note: Vacc'd= vaccinated individuals, Unvac= Unvaccinated individuals, All= burden of both vaccinated and unvaccinated individuals, YLL= years of life lost, YLD= YLD= years lived with disability, PICS= Post-Intensive care syndrome, Baseline= no permanent disability
